# Endovascular coil embolization for anomalous systemic artery supply to the lower lateral segment of the left lung: a case report

**DOI:** 10.3389/fmed.2026.1824390

**Published:** 2026-04-28

**Authors:** Chuan Shao, Liang Zhang, Yibing He, Yu Zhang

**Affiliations:** 1 Department of Respiratory and Critical Care Medicine, Ningbo Medical Center Lihuili Hospital, Ningbo, China; 2 Health Science Center, Ningbo University, Ningbo, China

**Keywords:** anomalous systemic artery supply, congenital vascular anomaly, digital subtraction angiography, hemoptysis, interventional embolization

## Abstract

A 40-year-old female who presented with “hemoptysis for 3 days” was referred to our hospital due to suspected bronchial artery malformation. Physical examination revealed no obvious abnormalities. Aortic CT angiography with three-dimensional reconstruction showed the descending aorta had an abnormal branch supplying the lower lateral segment of the left lung. Left pulmonary artery angiography under DSA showed poor filling of the lower lateral segment artery of the left lung, and aortography revealed an abnormal branch originating from the lower segment of the thoracic aorta supplying the left lower lateral segment. The diagnosis of anomalous systemic artery supply to the lower lateral segment of the left lung was confirmed. After angiography, a microcatheter was used to select the abnormal vessel, and coils were placed to embolize the abnormal artery. Re-angiography after embolization showed that the distal vessel disappeared. The patient had no obvious discomfort or complications during or after the procedure. Two months later, contrast-enhanced CT scan showed that the shadow of the left lower lobe had significantly absorbed, the coils were in place as expected, and the abnormal blood vessel was blocked well.

## Introduction

Abnormal systemic artery supply to the lower lateral segment of the left lung is a rare congenital vascular malformation, which refers to the abnormal systemic artery originated from the descending aorta supplying the lower lateral segment of the left lung. The pulmonary artery in this area is often absent, while the bronchus and lung parenchyma are normal.

We report a case of abnormal arterial supply to the lower lateral segment of the left lung. The CT angiography of the thoracic aorta with three-dimensional reconstruction clearly shows the origin and course of the abnormal vessel. The diagnosis was confirmed under digital subtraction angiography (DSA), and interventional embolization treatment was successfully performed simultaneously. We hope that the reporting of this case can enhance clinicians’ understanding of this disease, and reduce missed diagnoses and misdiagnoses.

## Case report

A 40-year-old female patient was admitted to our hospital on August, 2025, due to “hemoptysis for three days.” The blood was bright red and approximately 10 mL per day in volume. She also had low-grade fever with a temperature around 37.4 °C but no chest tightness, shortness of breath, nausea, vomiting, or other discomforts. Chest CT scan at a local hospital showed a localized exudative shadow in the left lower lobe. After receiving antibiotic and hemostatic treatment, she did not experience further episodes of hemoptysis. The CT angiography of the bronchial arteries indicated obvious dilation of the left bronchial artery originating from the descending aorta, recommending further evaluation with bronchial arteriography. Then the patient was referred to our hospital for further treatment. There were no abnormal findings in her past medical history, personal history, or family history.

### Physical examination

The patient’s temperature was 37 °C, pulse rate was 71 beats per minute, respiratory rate was 16 breaths per minute, blood pressure was 105/53 mmHg. No positive findings were observed during the physical examination.

### Laboratory tests and auxiliary exams

**Figure 1:**
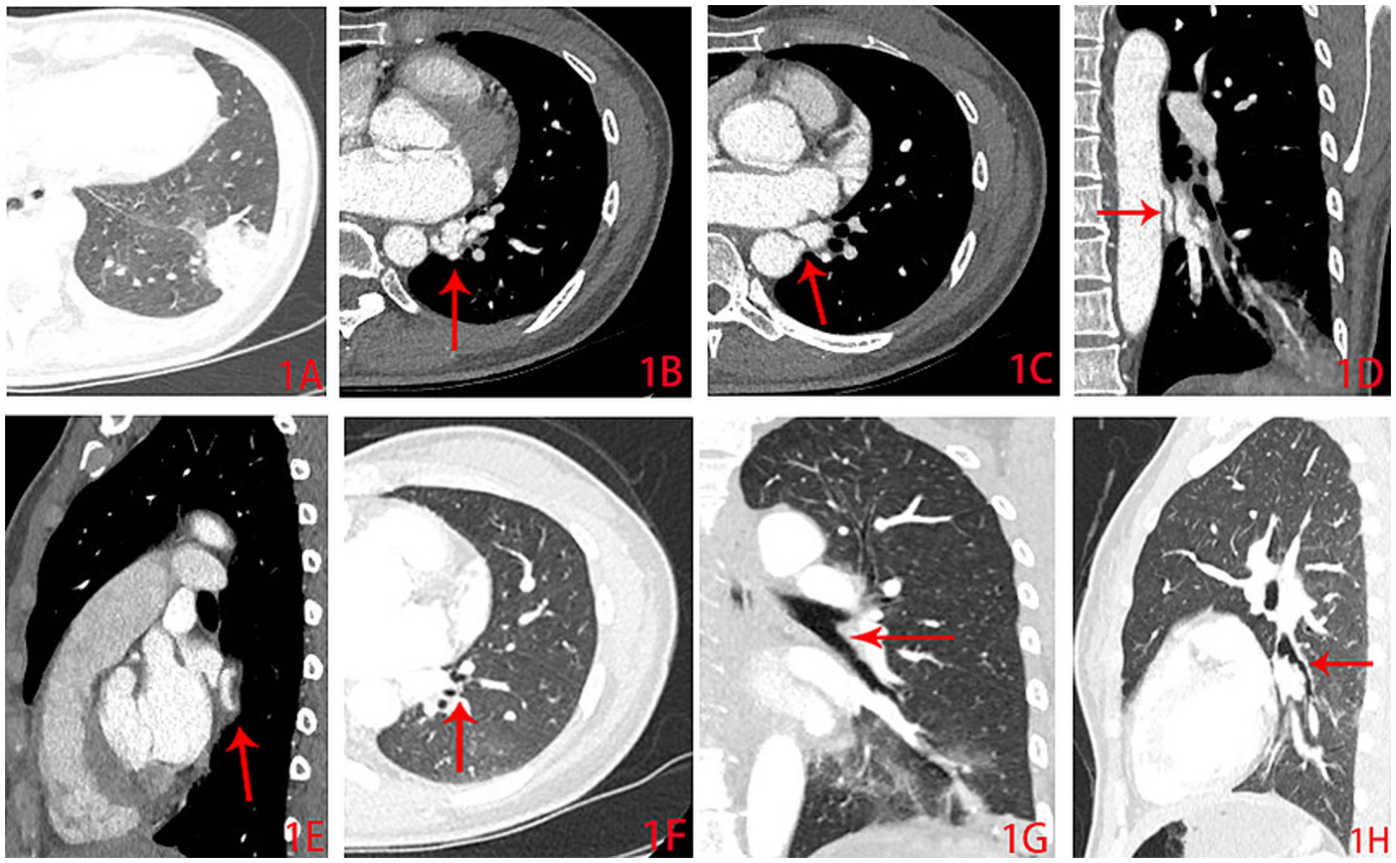
CT angiography images of the thoracic aorta (CTA). **(A)** The lung window of the CTA shows localized increase in density in the lower lateral segment of the left lung, with most areas showing consolidation and a small amount of ground-glass opacity; **(B,C)** The mediastinal window shows a dilated and tortuous vessel originating from the descending aorta; The arrows in **(C)** indicate the starting point of the abnormal arterial branch; **(D)** The coronal imaging shows the abnormal artery originating from the thoracic aorta and proceeding downward to participate in the blood supply of the left lower lung; **(E)** The abnormal artery partly shown in the sagittal image; **(F–H)** The bronchial tree structure of the lower lateral segment of the left lung was normal.

Routine blood test results showed a white blood cell count of 4.6 × 10^9^/L, neutrophil classification at 40.3%, hemoglobin level at 99 g/L, platelet count at 166 × 10^9^/L, and CRP at 4.4 mg/L. Erythrocyte sedimentation rate was 19 mm/h. Coagulation function and D-dimer levels were normal, while blood biochemistry tests indicated no abnormalities in liver or kidney function parameters, electrolytes, etc. An electrocardiogram (ECG) revealed no significant abnormalities. The aortic CTA scan indicated an abnormal branch originating from the descending aorta suppling the lower lateral segment of the left lung (Figure 1).

## Diagnosis and treatment

To confirm the diagnosis and provide treatment, transcatheter bronchial arteriography (TBA), pulmonary artery angiography (PA), and anomalous systemic arterial embolization were performed. The surgical procedure was as follows: The patient was placed in a supine position with electrocardiographic monitoring and oxygen inhalation. After routine disinfection and draping, local anesthesia at the puncture site was administered using 1% lidocaine. A modified Seldinger technique was applied to place a 5F vascular sheath into the right femoral vein for access, followed by another 5F sheath insertion in the right femoral artery.

**Figure 2:**
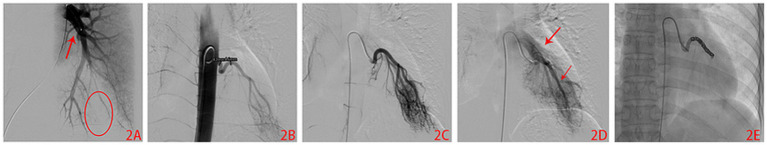
Angiography of the left pulmonary artery and thoracic aorta. **(A)** The pulmonary artery angiography shows a local blood supply defect in the lower left area, as indicated by the red circle. The arrow showed the descending branch of the left pulmonary artery; **(B)** The abnormal artery originated from the descending aorta proceeds downward before curving upward; **(C)** The abnormal artery supplying the lower lateral segment of the left lung; **(D)** The abnormal arteries’ venous blood flows back to the left pulmonary veins. The fine arrow showed the venous return of the lower lateral segment of the left lung, and the thick arrow showed the left inferior pulmonary vein; **(E)** After embolization, the distal blood vessels no longer show up.

A Pigtail catheter (0.035–150 cm) was introduced through the venous sheath and used to perform left pulmonary artery angiography. The results showed poor arterial filling in the lower lateral segment of the left lung (Figure 2A) and the left lower pulmonary vein does not take up contrast. Subsequently, systemic artery angiography was performed revealing an abnormal branch originated from the lower segment of the thoracic aorta supplying the lower lateral segment of the left lung (Figure 2B), and the area supplied by the abnormal vessel corresponded to the defect area shown in the pulmonary artery angiography. A 0.035–150 cm guidewire was used to introduce the SIM catheter, which was selectively placed at the lesion vessel. Selective angiography of the culprit vessel reveals the area with abnormal arterial supply (Figure 2C) and the venous blood in this area flows back to the left lower pulmonary vein (Figure 2D). The NESTE coils (18–14-6 × 1) and the tower coils (18S-2/6 × 7) were placed successively to embolize the abnormal systemic arterial branches. Re-angiography showed that the distal vessels were no longer visible (Figure 2E). The patient experienced no significant discomfort during or after the procedure and had no postoperative complications. To reduce local inflammation following embolization and prevent infection, she received intravenous dexamethasone of 10 mg daily for 3 days along with cefmetazole of 2 g twice-daily.

**Figure 3:**
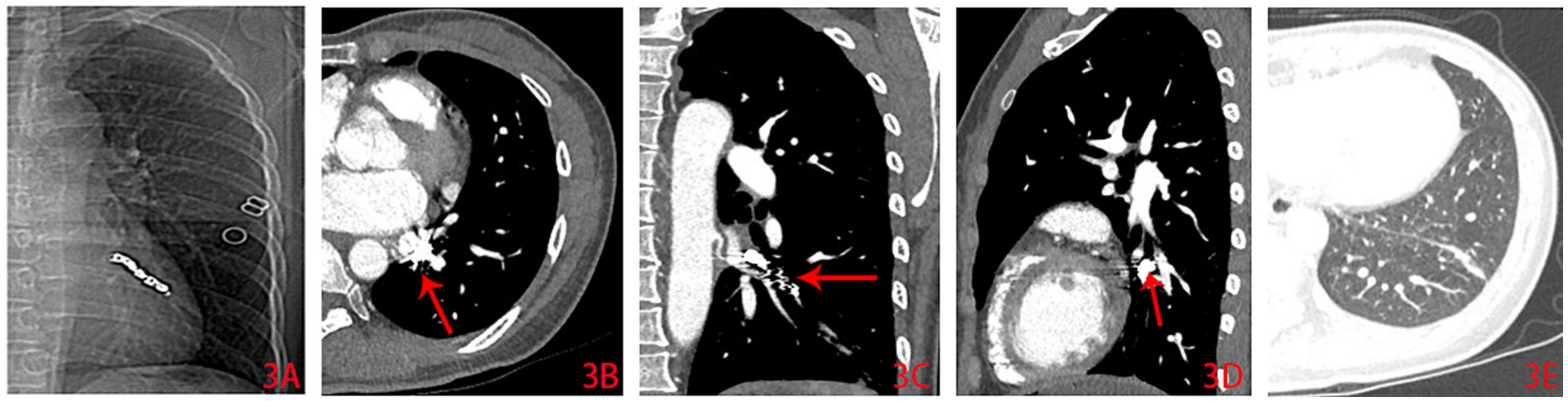
Contrast-enhanced chest CT images 2 months after embolization treatment. **(A)** The chest X-ray film shows the metallic shadow of the embolized coils; **(B)** the CT mediastinum window shows a high-density image of the metal coils; **(C)** the CT coronal view shows the abnormal blood vessel that have been embolized with coils; **(D)** the CT sagittal view shows the metal coils images in the abnormal artery; **(E)** 2 months after embolization treatment, the lung window shows that the shadow in the lower lateral segment of the left lung had significantly absorbed, with a few fibrous lesions left.

Two months after the surgery, the patient reported no obvious discomfort. The enhanced CT scan of the thoracic aorta showed that the coils were in good position (Figures 3A–D), and the exudative lesion in the left lower lobe had significantly absorbed (Figure 3E).

## Discussion

Anomalous systemic artery supply within pulmonary tissue can be categorized into two types: anomalous systemic arterial dilation and variants of the systemic arteries. The former is typically acquired and associated with conditions such as bronchiectasis or chronic inflammation, while the latter is congenital in nature and includes pulmonary sequestration and anomalous systemic circulation.

**Figure 4:**
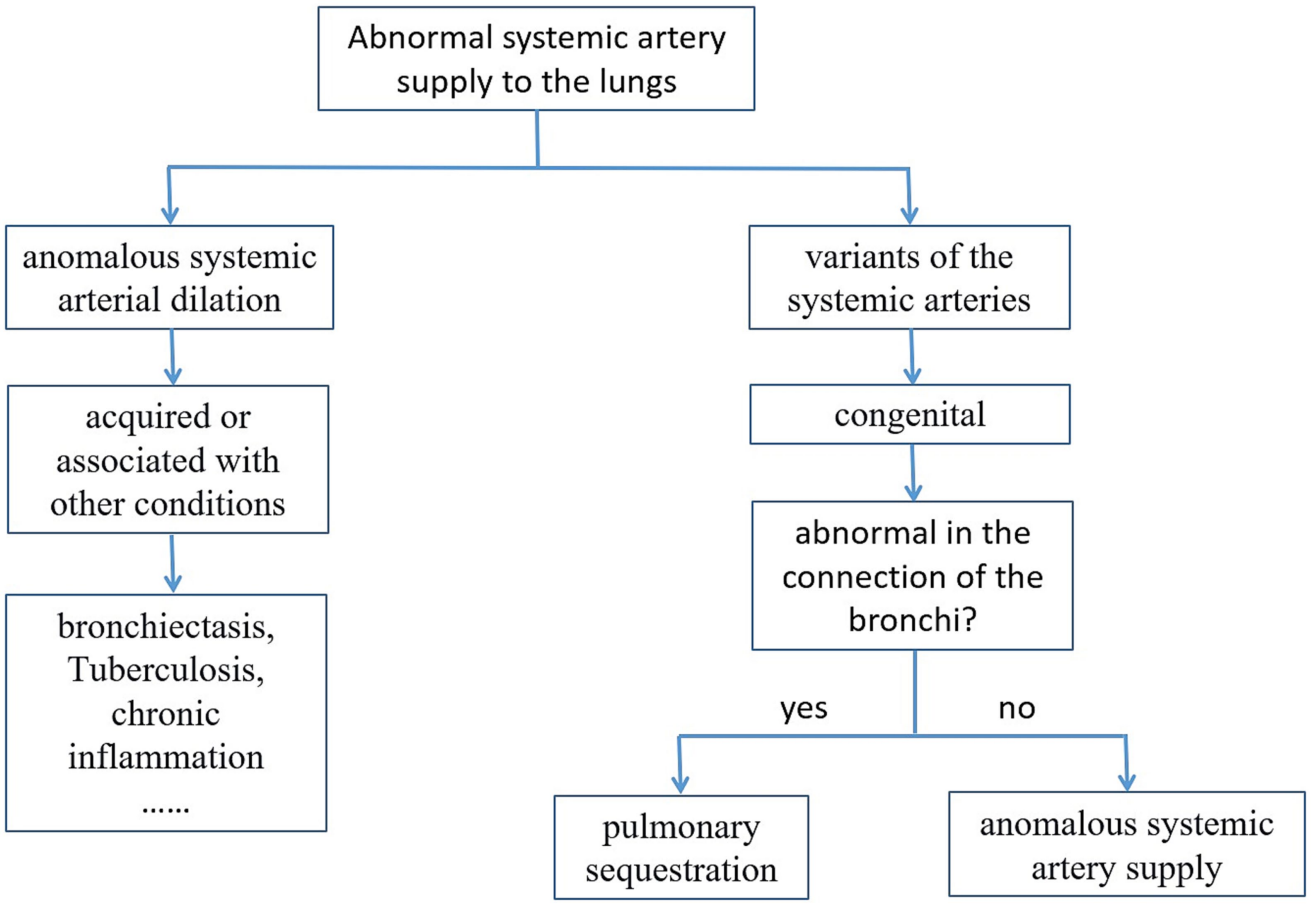
Diagnostic approach for anomalous systemic arterial supply to the lungs.

The lower lateral segment of the left lung is most commonly affected by these anomalies. Anomalous systemic supply to this specific lung segment represents a rare variant among pulmonary vascular developmental abnormalities. Historically, it has been associated with naming conflicts, such as being referred to as intrapulmonary sequestration (Pryce Type I) or anomalous pulmonary venous drainage in the absence of pulmonary sequestration (1). It is important to distinguish this condition from typical pulmonary sequestration. The key difference lies in the fact that affected pulmonary segments do not exhibit abnormal bronchial structures or lung parenchyma, which are characteristic features of pulmonary sequestration. The diagnostic approach for anomalous systemic arterial supply to the lungs is shown in Figure 4. In this case, the patient is a middle-aged female. She has no history of tuberculosis or bronchiectasis. The previous chest CT scan did not reveal any chronic inflammatory lesions in the lungs. Therefore, normal systemic artery dilation can be ruled out, and the possibility of congenital variant arteries is more likely. By carefully examining the images, it can be observed that the bronchial connection at the lesion site in the left lower lobe is normal. Thus, pulmonary sequestration can be excluded. Finally, through DSA, the diagnosis of abnormal systemic artery supplies the lower lateral segment of the left lung was confirmed.

The exact etiology remains unclear but is believed to be linked to embryonic vascular developmental anomalies. It may arise from incomplete regression of primitive systemic arterial branches during early development, resulting in abnormal connections between these vessels and the pulmonary vasculature. This process can lead to pulmonary artery hypoplasia or stenosis at the affected segment (2). Anatomically, anomalous systemic supply predominantly affects the left lower lobe’s basal segments with very rare involvement on the right side (3). The source of these anomalous arteries is most commonly found in the descending thoracic aorta but may also originate from intercostal arteries, coeliac trunk, subclavian artery, or internal mammary artery. While single-vessel anomalies are more common among patients, multiple-vessel involvement cases remain exceptionally rare. In most cases, the lung tissue involved does not receive blood supply from the pulmonary artery, which is referred to as the complete type; in some cases, there is a narrowed blood supply from the pulmonary artery, which is called the incomplete type (4). The lung tissue with abnormal systemic circulation blood supply has normal pulmonary vein drainage and bronchial connections. Pathologically, abnormal blood supply arteries are often characterized by thickened vessel walls, hyaline degeneration, atherosclerosis, etc. The affected lung segments may show intrapulmonary hemorrhage and hemosiderin deposition. This patient is a typical case. The abnormal artery is a single vessel, originating from the descending aorta and supplying the lower lateral segment of the left lung. The affected lung tissue lacks pulmonary artery supply and the refluxing pulmonary veins are normal.

The symptoms can occur at any age. The symptoms usually appear in young and middle-aged people. Some studies suggest that it is more common in the Asian population, with more men than women (5). The clinical manifestations are not specific. Hemoptysis is the most common symptom. The capillary beds in the affected lung area are exposed to a higher systemic blood pressure, making them prone to capillary rupture and bleeding, resulting in blood accumulation in the alveoli. Some patients experience an increase in left heart load due to left-to-right shunting, which subsequently leads to pulmonary hypertension and ultimately results in an increase in right heart load, manifesting as congestive heart failure (6). Additionally, the presence of large abnormal arterial branches and the compression of dilated pulmonary veins, which cause incomplete obstruction of adjacent bronchi, can lead to recurrent infections (4). The severity of the symptoms is usually related to the pressure and diameter of the abnormal blood vessels. Some patients may have no symptoms at all due to a small blood flow. The hemoptysis of this patient was the first occurrence and there were no other symptoms. The local high-density shadow displayed in the lung window of the chest CT is considered to be related to the accumulation of blood in the alveolar cavities. After embolization treatment, the lesion in the left lower lobe absorbed rapidly and significantly. Therefore, the clinical manifestations, imaging findings and treatment outcome of this case were typical and fully conformed to the characteristics of abnormal systemic arterial supply to the lungs.

The diagnosis of this disease mainly relies on imaging techniques. Chest X-rays may show nodular or mass-like shadows behind the heart shadow. However, due to the limitation of overlapping images of the chest X-ray, it is prone to result in misdiagnosis. In CT scan without contrast enhancement, dilated blood vessels are prone to be mistaken for nodules or occupying lesions. Multi-row spiral CT scan with contrast enhanced, combined with three-dimensional reconstruction, has a high diagnostic value, and it can clearly and intuitively display the origin, shape and course of the abnormal arteries. Enhanced CT scan can show that the pulmonary arteries in the affected area suddenly become narrow or disappear, while the pulmonary vein drainage remains normal. The affected lung structure may show no obvious abnormalities, or it may present as a reduction in lung volume, with local density increase manifested as ground-glass opacities or consolidation shadows, which may be related to pulmonary alveolar hemorrhage or pulmonary congestion. DSA is the gold standard for diagnosis. Transcatheter abnormal systemic arterial angiography combined with pulmonary artery angiography can determine the origin of the abnormal arterial branches and the blood supply status of the affected lung tissue. At the same time, interventional embolization therapy can be performed. Cardiac color Doppler ultrasound, as an auxiliary diagnostic tool, can be used to assess pulmonary artery pressure, tricuspid regurgitation and pulmonary regurgitation, as well as ventricular diameter, etc.

The differential diagnoses for this disease include pulmonary intra-lobar sequestration and systemic arterial dilatation, for example as a result of an infection or a tumor (as shown in Figure 4). The affected area in pulmonary sequestration is the non-functional lung tissue, presenting as solid, cystic-solid or cystic lesions with abnormal systemic blood supply. The lower lobe of the left lung is the most commonly affected area. Abnormal blood supply arteries can originate from the descending aorta, intercostal arteries, or subclavian arteries, and return through the pulmonary veins. The absence of connection between the lesion and the bronchus is the main distinguishing feature of this disease. Dilation of systemic arteries is commonly observed in patients with chronic inflammatory diseases of the lungs, such as bronchiectasis, tuberculosis, and chronic obstructive pulmonary disease. Chronic inflammatory stimulation can cause vascular abnormalities. In these patients, anastomosis can form between the systemic and pulmonary arteries, with the most common being the anastomosis between the peripheral branches of the bronchial artery and the pulmonary artery. CT scan can display the images related to chronic inflammation. In this case, the patient initially visited a local hospital and underwent CT angiography of the bronchial arteries, which revealed abnormal blood vessels. However, no three-dimensional reconstruction was performed to carefully observe the vessel course and the bronchial condition of the corresponding lung tissue, nor was the patient’s previous lung CT images reviewed. As a result, the patient was mistakenly diagnosed as having a bronchial artery dilatation. Therefore, for patients in the clinical settings where there is a suspicion of abnormal arterial supply of the lung, a comprehensive analysis should be conducted based on the vessel course in the CTA, the morphology of the lung lesions, and bronchial drainage, and results of DSA, so as to achieve a confirmed diagnosis.

At present, there is no consensus on the appropriate timing for treatment and the best treatment methods for this disease. Surgical operations and interventional embolization are the commonly used treatment methods in previous case reports (6). Surgical treatment has shifted from traditional open thoracic surgery to minimally invasive thoracoscopic surgery, including surgical methods such as lobectomy, segmentectomy, arterial ligation, and arterial anastomosis (6). Transcatheter arterial embolization (TAE) has certain advantages in this disease and is the most commonly used treatment method among the cases reported in recent years. As a minimally invasive interventional therapy, it has the advantages of lower surgical risk, better patient tolerance, and faster recovery. Spring coils are the most commonly used embolic materials. Embolization of abnormal arteries reduces the pressure on the pulmonary vascular bed by blocking or reducing blood flow, corrects left-to-right shunting, reduces cardiac load, and preserves normal lung tissue. The potential complications of embolization therapy mainly include post-embolization syndrome and chest discomfort. A small number of patients may develop local pulmonary infarction after surgery, which usually resolves spontaneously within a short period of time (5).

## Conclusion

Anomalous systemic artery supply to the lower lateral segment of the left lung is a rare congenital disorder, characterized by an abnormal arterial supply originating from the descending aorta to the lower lateral segment of the left lung, accompanied by the absence or stenosis of the pulmonary artery in the corresponding segment, while the bronchus in the affected region is normal. Multi-slice spiral CT enhanced scanning and three-dimensional reconstruction are helpful for diagnosis, which should be confirmed by DSA finally. It is necessary to differentiate the disease from pulmonary sequestration. Observing the arterial supply, venous drainage, lung parenchyma and bronchial conditions is the key point for differentiation. The interventional embolization of the abnormal artery is highly efficient, convenient, with controllable risks and good patient tolerance, and thus can be used as an optimal treatment option.

## Data Availability

The original contributions presented in the study are included in the article/Supplementary material, further inquiries can be directed to the corresponding author.
