# Supplementary figures and images for: Endovascular coil embolization for anomalous systemic artery supply to the lower lateral segment of the left lung: a case report
Source: Front Med (Lausanne). 2026 Apr 28;13:1824390. doi: 10.3389/fmed.2026.1824390 (PMC13161108; doi:10.3389/fmed.2026.1824390)

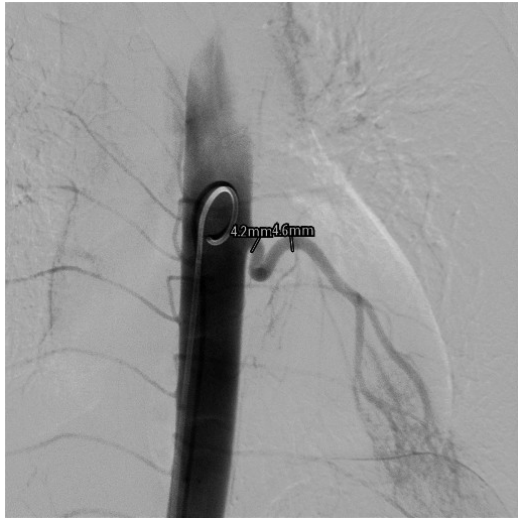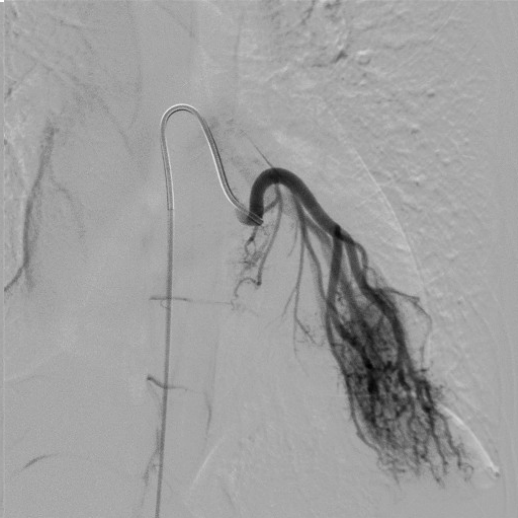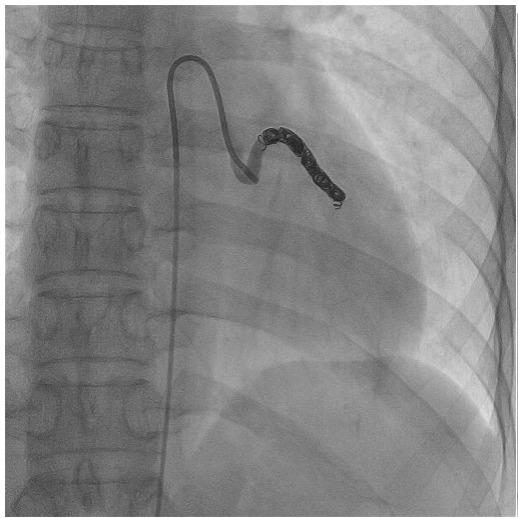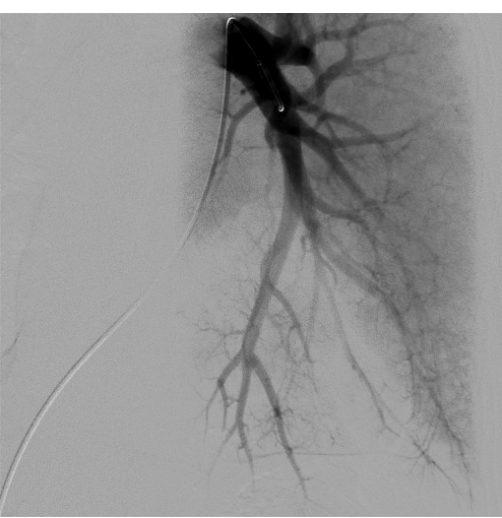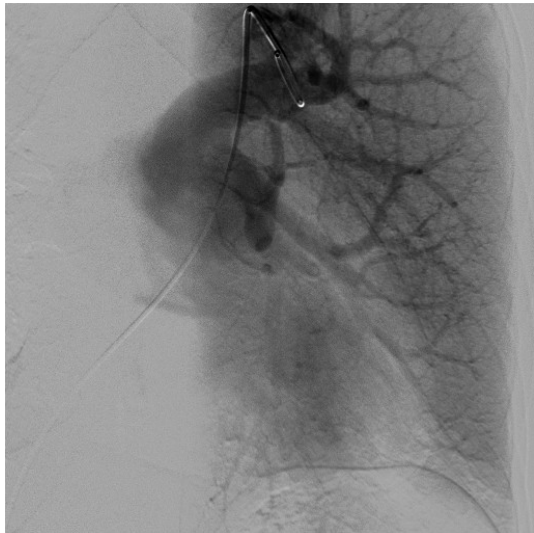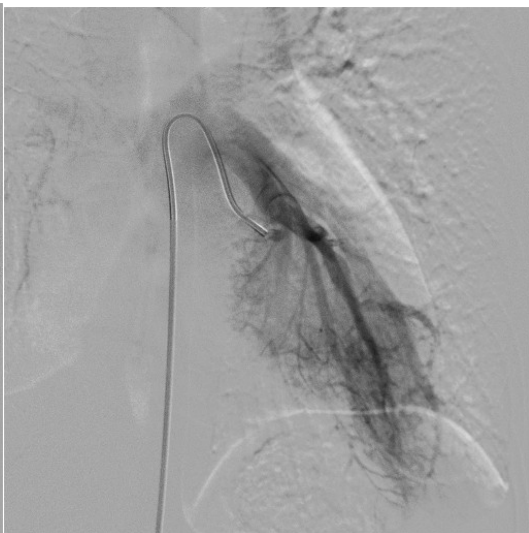

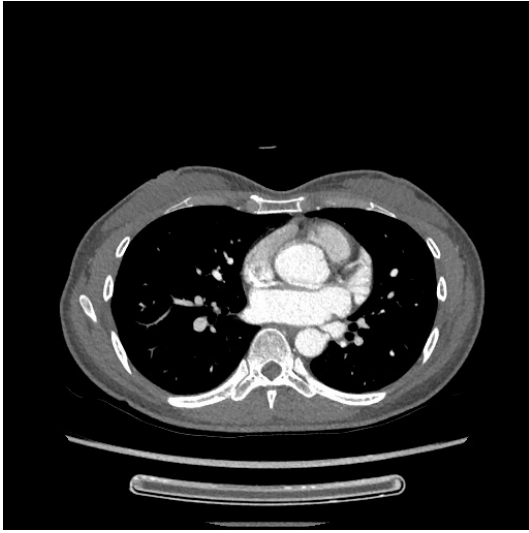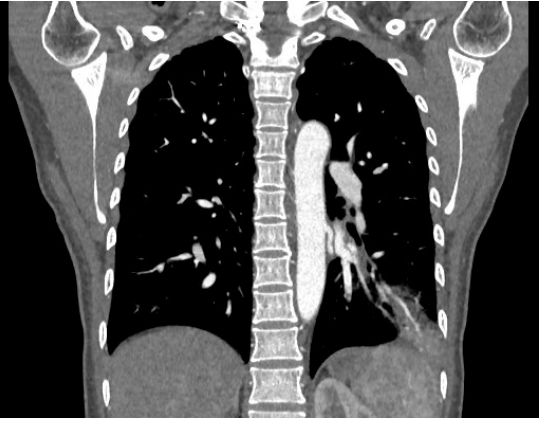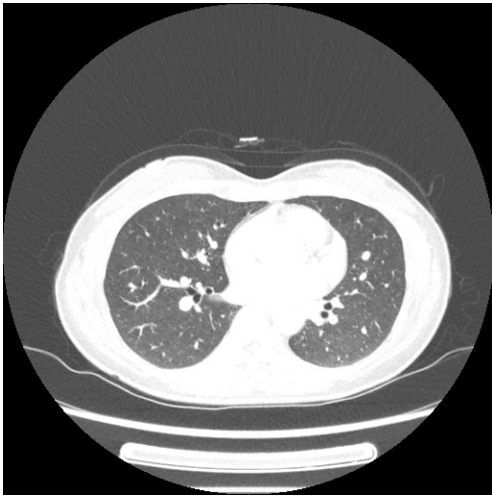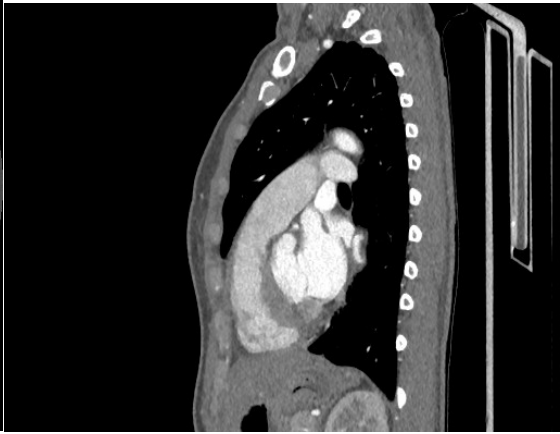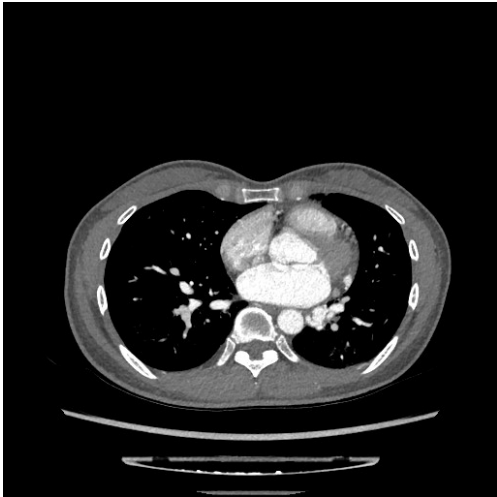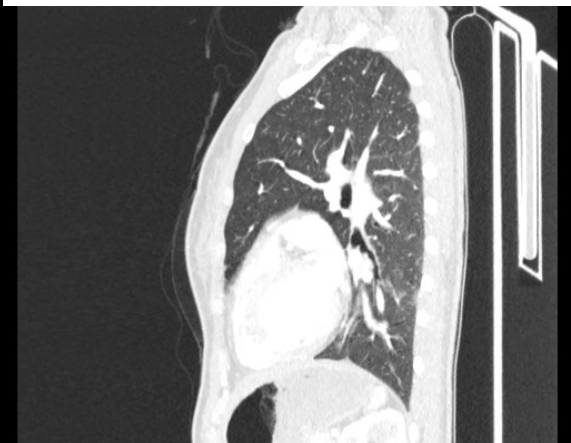

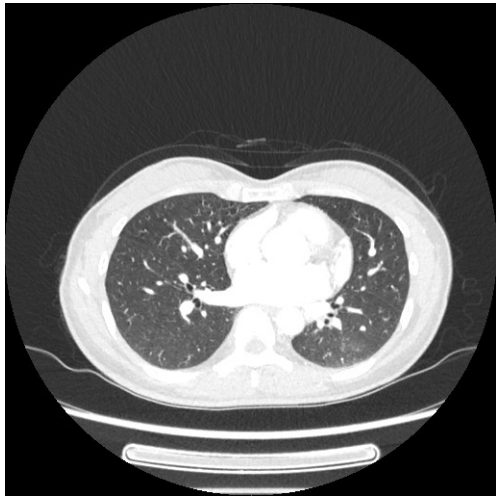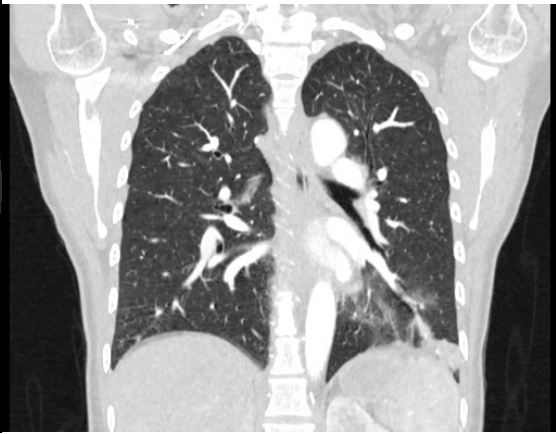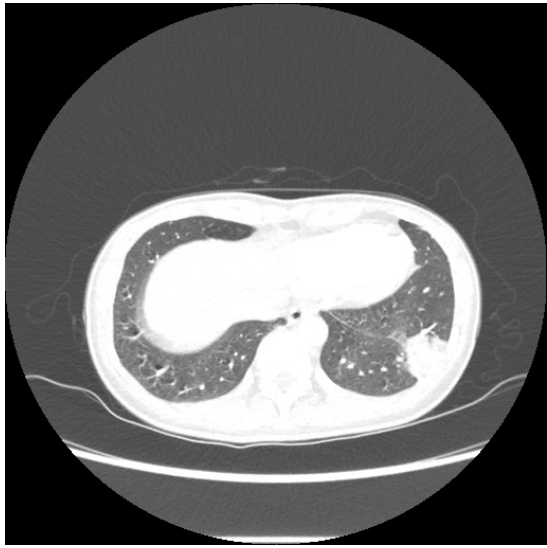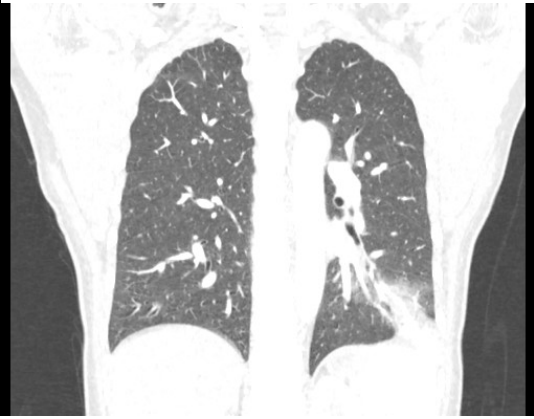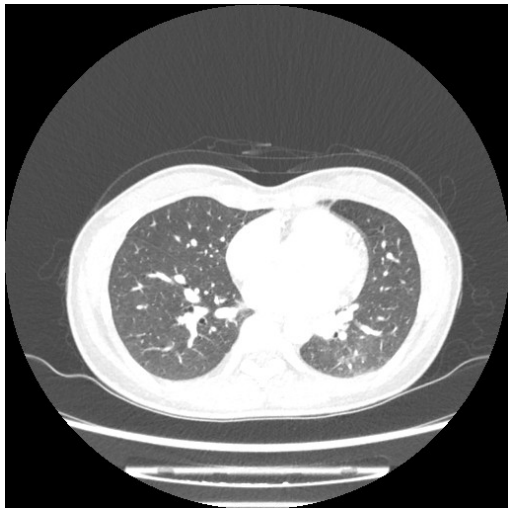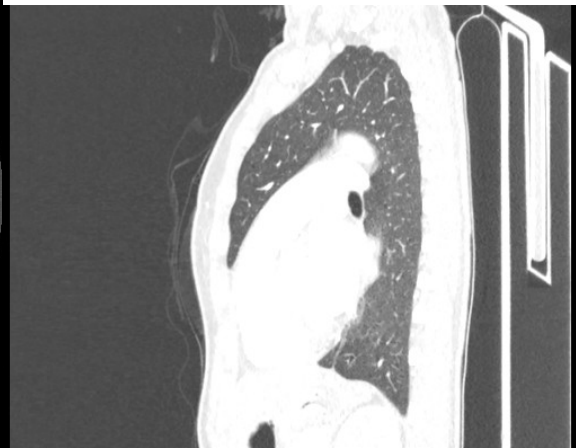

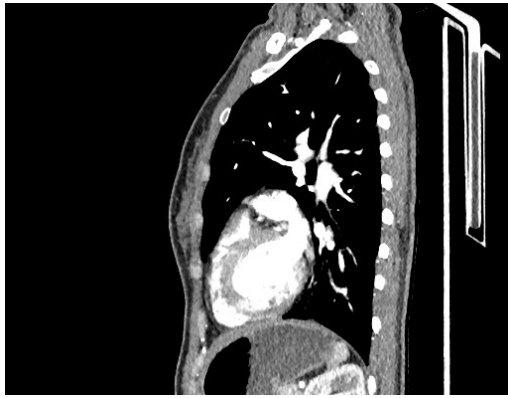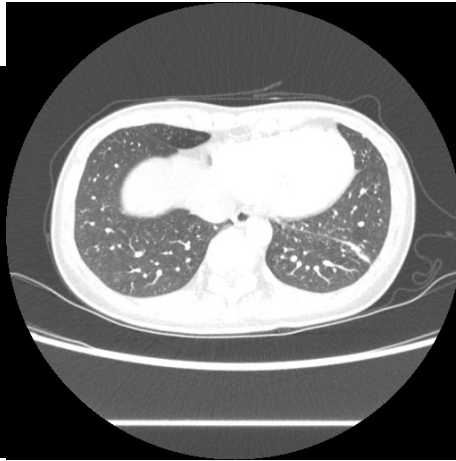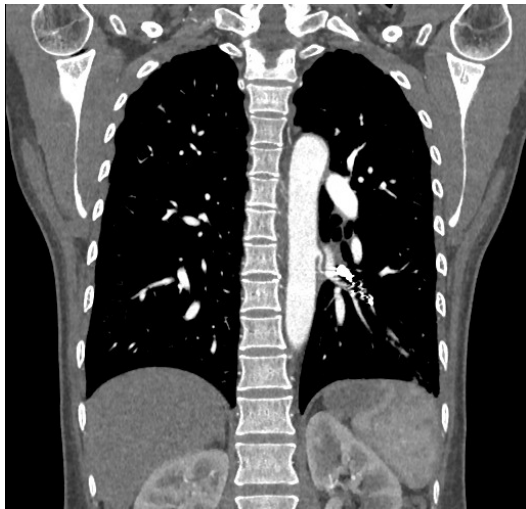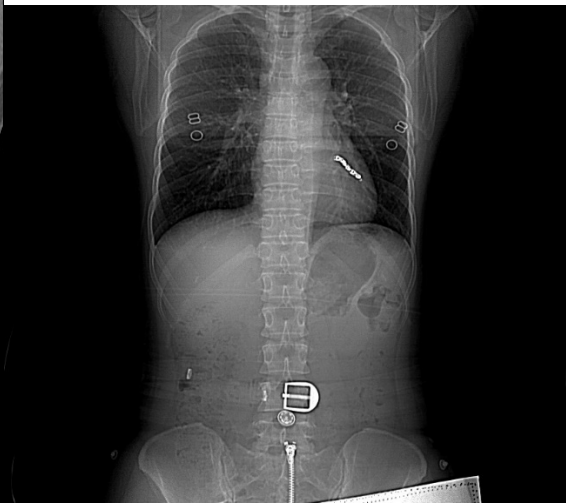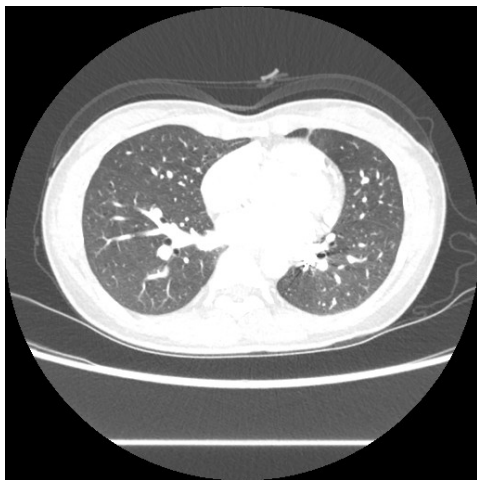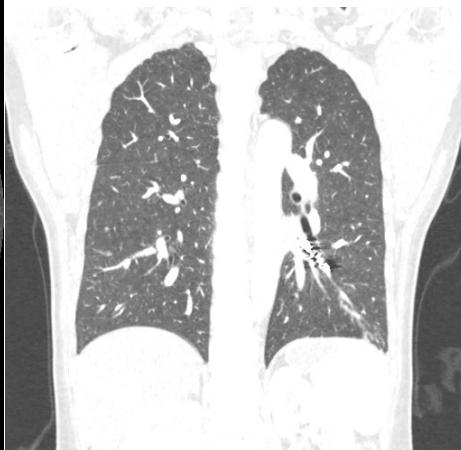

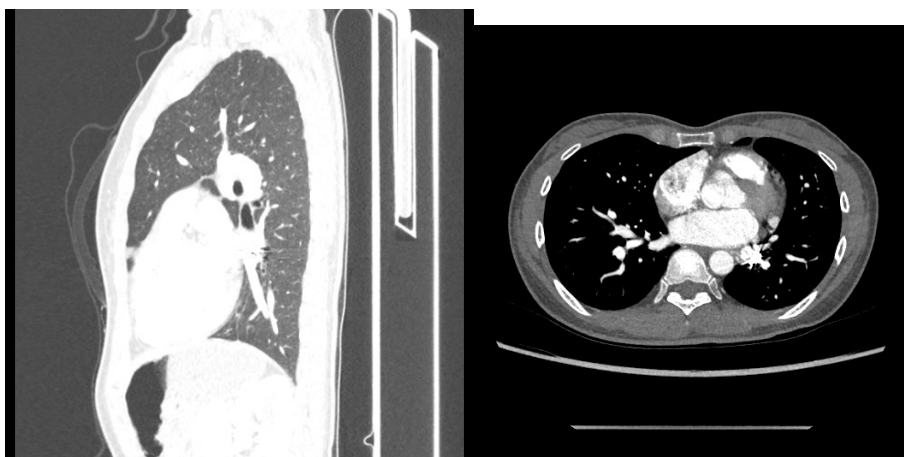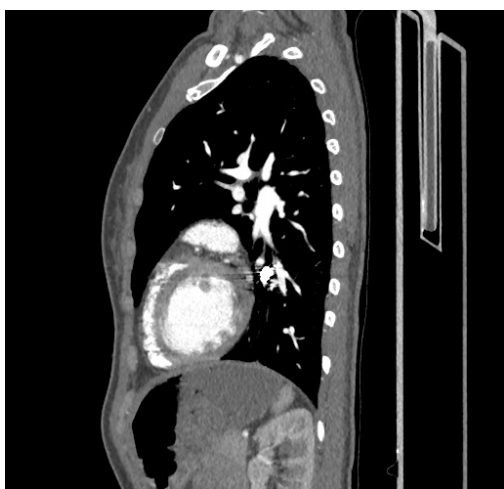

Supplement: Supplementary file 1 [file Data_Sheet_1.PDF]
